# Transcriptome profile analysis of leg muscle tissues between slow- and fast-growing chickens

**DOI:** 10.1371/journal.pone.0206131

**Published:** 2018-11-07

**Authors:** Pengfei Wu, Guojun Dai, Fuxiang Chen, Lan Chen, Tao Zhang, Kaizhou Xie, Jinyu Wang, Genxi Zhang

**Affiliations:** 1 College of Animal Science and Technology, Yangzhou University, Yangzhou, Jiangsu, China; 2 Key Laboratory for Animal Genetics, Breeding, Reproduction and Molecular Design of Jiangsu Province, Yangzhou, Jiangsu, China; University of Maryland Center for Environmental Science, UNITED STATES

## Abstract

Chicken is widely favored by consumers because of some unique features. The leg muscles occupy an important position in the market. However, the specific mechanism for regulating muscle growth speed is not clear. In this experiment, we used Jinghai yellow chickens with different body weights at 300 days as research subjects. The chickens were divided into fast- and slow-growing groups, and we collected leg muscles after slaughtering for use in RNA-seq. After comparing the two groups, 87 differentially expressed genes (DEGs) were identified (fold change ≥ 2 and FDR < 0.05). The fast-growing group had 42 up-regulated genes and 45 down-regulated genes among these DEGs compared to the slow-growing group. Six items were significantly enriched in the biological process: embryo development ending in birth or egg hatching, chordate embryonic development, embryonic skeletal system development, and embryo development as well as responses to ketones and the sulfur compound biosynthetic process. Two significantly enriched pathways were found in the KEGG pathway analysis (P-value < 0.05): the insulin signaling pathway and the adipocytokine signaling pathway. This study provides a theoretical basis for the molecular mechanism of chicken growth and for improving the production of Jinghai yellow chicken.

## Introduction

Chicken is widely favored by consumers because of its delicate meat quality, delicious taste and rich nutrition as well as the ease of cooking. The demand for chicken is increasing in recent years. However, the mechanism for regulating the growth of chicken is not clear. Chicken growth traits are controlled by multiple genes and some genes were also shown to be related to growth, including MSTN [1], MYOD [2], MYOG [3]. Heritability estimates suggested that the genetics of chickens could be improved [4, 5], and the growth rate of broilers has been also greatly increased in recent decades [6].

Transcriptomics are the basis for gene structure and function research [7]. The complete set of transcripts is known as a general transcriptome, including protein-coding messenger RNA (mRNA) and non-coding RNA [ncRNA: ribosomal RNA (rRNA), transfer RNA (tRNA), and other ncRNAs [8, 9]. The narrow sense of the transcriptome mainly refers to all mRNA. Transcriptional sequencing (RNA-seq) has developed rapidly in recent years, which is a technique for analyzing the transcriptome by deep sequencing technology [10]. The whole transcriptome was detected at the single nucleotide level with the technology. RNA-seq can analyze the structure and expression level of the transcript, and it becomes an important means of gene expression and transcriptional analysis [11, 12]. In recent years, RNA-seq has been widely used in research on livestock and poultry transcriptomes. Woncheoul Park et al. [13] performed RNA sequencing (RNA-seq) using the kidneys of broiler chickens fed diets containing three different concentrations of Ca (0.8%, 1.0%, and 1.2%) and they found 128, 141, and 103 DEGs between different concentrations (0.8 and 1.0, 0.8 and 1.2, and 1.0 and 1.2% Ca). Pathak SK et al. [14] collected blood from crossbred and indigenous (desi) piglets for RNA-seq both on the day of and 4 weeks after vaccination against classical swine fever (CSF). To investigate goose immune-related genes, Wang et al. [15] performed deep transcriptome and gene expression analyses of spleen samples using paired-end sequencing technology (Illumina).

The production performance of livestock and poultry reflects the status of animal growth and development. Generally, the production performance includes two parts: growth performance and slaughter performance. The weight of the leg muscle is an important index for determining the slaughter performance of broilers. In the experiment, we took Jinghai yellow chickens with different body weights at 300 days as the research subjects. The chickens were divided into fast- and slow-growing groups (high body weight and low body weight). We selected leg muscles after slaughtering and used then for RNA-seq. Finally, we screened differentially expressed genes and the corresponding enriched pathways related to growth through a bioinformatics analysis. The results provide a theoretical foundation for revealing the molecular mechanism of the growth for chickens and improving the production performance of the Jinghai yellow chicken.

## Materials and methods

### Ethics statement

The experiments were fully consistent with the codes made by the Chinese Ministry of Agriculture. The animal experiments performed in the study were all evaluated and approved by the Animal Ethics Committee of Yangzhou University.

### Animals and tissues

The chickens used in this study were Jinghai yellow chickens. They were obtained from Jiangsu Jinghai Poultry Industry Group Co., Ltd. (Nantong City, Jiangsu Province, China). Chickens were raised on the ground at 0–16 weeks of age and were transferred into cages after 16 weeks. Artificial illumination was used during the whole process. The birds had access to feed and water ad libitum. Three fast-growing and three slow-growing female chickens with similar weights at the age of 300 days were selected from the Jinghai yellow chicken population. We firstly used the Xylazine Hydrochloride(SIGMA, X-1251) to anesthetize the chickens according to the amount of 8 mg/kg. When the feathers on both wings and tails fall down and finally failed to respond to stimuli, it shows that the chickens have been completely anaesthetized. And then they were all sacrificed with bleeding of carotid artery. We recorded the live weight and leg muscle weight of each chicken for the analysis of significant difference. The leg muscles were then collected immediately, snap-frozen in liquid nitrogen, and stored at −80°C until RNA extraction.

### Total RNA extraction and RNA library preparation

The method of extracting RNA leg muscles was from Xue et al. [16]. RNA degradation and contamination were monitored on 1% agarose gels. The purity, concentration and integrity of the RNA were checked using the NanoPhotometer spectrophotometer (IMPLEN, CA, USA), Qubit RNA Assay kit in Qubit 2.0 Flurometer (Life Technologies, CA, USA) and the RNA Nano 6000 Assay kit of the Agilent Bioanalyzer 2100 system (Agilent Technologies, CA, USA), respectively. Sequencing libraries were generated using the NEBNext UltraTM RNA Library Prep Kit for Illumina (NEB, USA) according to the manufacturer’s recommendations, and index codes were added to attribute the sequences to each sample. The PCR products were purified (AMPure XP system, Beckman Coulter, Beverly, USA), and the library quality was assessed using the Agilent Bioanalyzer 2100 system (Agilent Technologies, CA, USA).

### Clustering and sequencing

Our sequencing project was conducted by the Biomarker Technologies company (http://www.biomarker.com.cn/). The clustering of the index-coded samples was performed on a cBot Cluster Generation System using a TruSeq PE Cluster kit v4-cBot-HS (Illumia) according to the manufacturer’s instructions. After cluster generation, the library preparations were sequenced on an Illumina Hiseq 2500 platform, and paired-end reads were generated. The read length is 100bp. Finally, the raw data was uploaded to the NCBI Sequence Read Archive and the accessions of SRA for the submission is SRP144529.

### Statistical analysis

The software SPSS 13.0 was used to analyze the difference in the live weight and leg weight between the slow- and fast-growing groups. The independent sample’s t test was used to compare means.

### Quality control and comparative analysis

The raw data (raw reads) in fastq format were first processed using in-house Perl scripts. Using this step, clean data (clean reads) were obtained by removing reads containing adapters, reads containing poly-N and reads of low-quality. At the same time, the Q20, Q30, GC-content and sequence duplication level of the clean data were calculated. All the downstream analyses were based on high-quality clean data.

The adaptor sequences and low-quality sequence reads were removed from the data sets. The raw sequences were transformed into clean reads after data processing. These clean reads were mapped to the reference genome sequence (Galgal4). Only reads with a perfect match or one mismatch were analyzed and annotated based on the reference genome. Tophat2 [17, 18] was used to map the reads to the reference genome.

### Differential expression analysis

The quantification of gene expression levels was performed as follows. The gene expression levels were estimated according to fragments per kilobase of transcript per million fragments mapped (FPKM) [19]. Differential expression analysis between the two groups was performed using the DESeq R package [20]. DESeq provides statistical routines for determining differential expression in digital gene expression data using a model based on the negative binomial distribution. The genes with an adjusted P-value ≤ 0.05 and a fold change ≥ 2 found by DESeq were considered differentially expressed. Fold change represents the ratio of the expression between the two groups. The resulting P-values were adjusted using Benjamini and Hochberg’s approach for controlling the false discovery rate.

### GO and KEGG pathway enrichment analysis

The Gene Ontology database [21, 22] (GO: http://www.geneontology.org/) is a structured, standard biological annotation system built in 2000 by an organization (Gene Ontology Consortium), and it aims at establishing a standard vocabulary systematic knowledge of genes and their products. KEGG [23, 24] (http://www.genome.jp/kegg/) is a database resource for understanding high-level functions and utilities of the biological system, including the cell, the organism and the ecosystem, from molecular-level information, especially large-scale molecular datasets generated by genome sequencing and other high-throughput experimental technologies. All the target genes of the differentially expressed mRNA were subjected to Gene Ontology (GO) and KEGG pathway enrichment analysis by using the DAVID 6.7 Functional Annotation Tool [25] (http://david.abcc.ncifcrf.gov/).

### Verification of RNA-seq results using qRT-PCR

The other 6 Jinghai yellow chickens at the age of 300 days were choosen and divided into two groups according to their body weight. Collect the leg muscles and extract RNA for verification of RNA-seq results using qRT-PCR. The total RNA was isolated from the leg muscle tissue of Jinghai yellow chickens, and mRNA was reverse transcribed into cDNA using the PrimeScript RT Master Mix (Perfect Real Time) kit (TaKaRa Biotechnology Co Ltd, Dalian, China). The primers used for quantification in the study were designed using Primer-BLAST on the NCBI website (https://www.ncbi.nlm.nih.gov/tools/primer-blast/). To avoid the effects of genomic DNA, primers must be separated by at least one intron in the corresponding gene. In the study, β-actin was used as the housekeeping gene [16, 26]. The number of replications for each sample is three. The qPCR was conducted on an Applied Biosystems 7500 real-time PCR system (Applied Biosystems) in a total volume of 20 μL with 10 μL of SYBR® Premix Ex Taq (2×), 0.4 μL of ROX Reference Dye II (TaKaRa Biotechnology Co Ltd, Dalian, China), 0.4 μL of each primer (10 μM), 6.8 μL of RNase-free water and 2 μL of cDNA. The cycling parameters were 95°C for 30 s, followed by 40 cycles of 95°C for 5 s and 60°C for 34 s. Melting curve analyses were performed following the amplifications. The quantification of gene expression was performed using the comparative threshold cycle (2^−ΔΔCT^) method [27].

## Results

### Comparison of the live weight and leg muscle weight between the two groups

The least squares mean between the slow- and fast-growing groups selected for RNA sequencing are presented in Table 1. Chickens in the fast-growing group had a higher live weight than those in the slow-growing group (P = 0.000 < 0.001). A significant difference (P = 0.012 < 0.05) in leg muscle weight between the two groups was also found.

**Table 1:** Analysis of differences between the two groups.

Traits	Low weight	High weight
Live weight(g)	1353.33±14.53^A^	2553.33±97.31^B^
Leg muscle weight(g)	94.27±2.27^a^	182.73±10.74^b^

**Note:** Means in the same row with different lowercase letters indicate significant differences (P < 0.05), with different capital letters also indicating significant differences (P < 0.01).

### Sequencing data and quality control

After the transcriptional analysis of the 6 samples, 30.78Gb clean data was obtained by the quality control. The clean data from each sample reached 4.52Gb, the GC content reached between 52.17% to 53.68%, and the percentage of Q30 base in each sample was greater than 80.09% (Table 2). The above results indicate that the data could be used for further analysis.

**Table 2:** Sequencing data.

Samples	Clean reads	Clean bases	GC Content	%≥Q30
T1	25,684,671	5,187,451,860	52.17%	80.15%
T2	27,925,040	5,639,352,036	53.68%	80.30%
T3	23,379,518	4,721,718,610	52.55%	80.09%
T4	22,362,504	4,516,161,206	53.60%	81.13%
T5	27,526,111	5,559,154,066	52.38%	80.24%
T6	25,524,976	5,154,259,598	53.02%	81.29%

**Note:** Clean reads: total number of pair-end reads in the clean data; Clean bases: total number of bases in the clean data; GC content: percentage of G and C bases in the clean data; % ≥ Q30: the percentage of Q30 base.

### Comparative analysis

The comparison efficiency of the total reads compared to the reference genome of the 6 samples was between 72.50% and 74.42%, and the percentage of reads compared to the only location of the reference genome was 69.59% to 72.16% (Table 3) in the clean reads. This indicated that the results were reliable. In the successfully compared reads, 68.68% to 70.15% were in exon, 10.01% to 10.59% were in introns, and 19.65% to 20.99% were intergenic. In theory, the reads from the mature mRNA should map to the exons, but this was not the reality. We thought that reads mapping to the intron may be due to the mRNA precursors and the intron reservations with variable shear. This result could also map to the intergenic region because the genome annotation is not perfect.

**Table 3:** Comparison results.

Sample	Total Reads	Mapped Reads	Uniq Mapped Reads	Multiple Mapped Reads	Reads Mapped to ‘+’	Reads Mapped to ‘-‘
T1	51,369,342	37,735,076 (73.46%)	36,331,091 (70.73%)	1,403,985 (2.73%)	18,795,619 (36.59%)	18,654,167 (36.31%)
T2	55,850,080	40,489,093 (72.50%)	38,865,457 (69.59%)	1,623,636 (2.91%)	20,142,523 (36.07%)	20,014,964 (35.84%)
T3	46,759,036	34,530,849 (73.85%)	33,188,302 (70.98%)	1,342,547 (2.87%)	17,206,777 (36.80%)	17,085,774 (36.54%)
T4	44,725,008	32,646,136 (72.99%)	31,635,623 (70.73%)	1,010,513 (2.26%)	16,263,304 (36.36%)	16,153,912 (36.12%)
T5	55,052,222	40,970,657 (74.42%)	39,726,232 (72.16%)	1,244,425 (2.26%)	20,439,448 (37.13%)	20,286,215 (36.85%)
T6	51,049,952	37,953,225 (74.35%)	36,622,732 (71.74%)	1,330,493 (2.61%)	18,885,017 (36.99%)	18,787,799 (36.80%)

**Note:** Total Reads: the number of single-end reads in the clean data; Mapped Reads: the number of reads on the reference genome and the percentage of mapped reads in the clean reads; Uniq Mapped Reads: the number of reads compared to the only location of the reference genome and the percentage of clean reads; Multiple Map Reads: the number of reads compared to the multiple locations of the reference genome and the percentage of multiple map reads in the clean reads; Reads Map to ‘+’: the number of reads compared to the positive-strand and the percentage of clean reads. Reads Map to Reads Map to ‘-‘: the number of reads compared to the negative-strand and the percentage of clean reads.

### Differentially expressed genes

**Fig 1:**
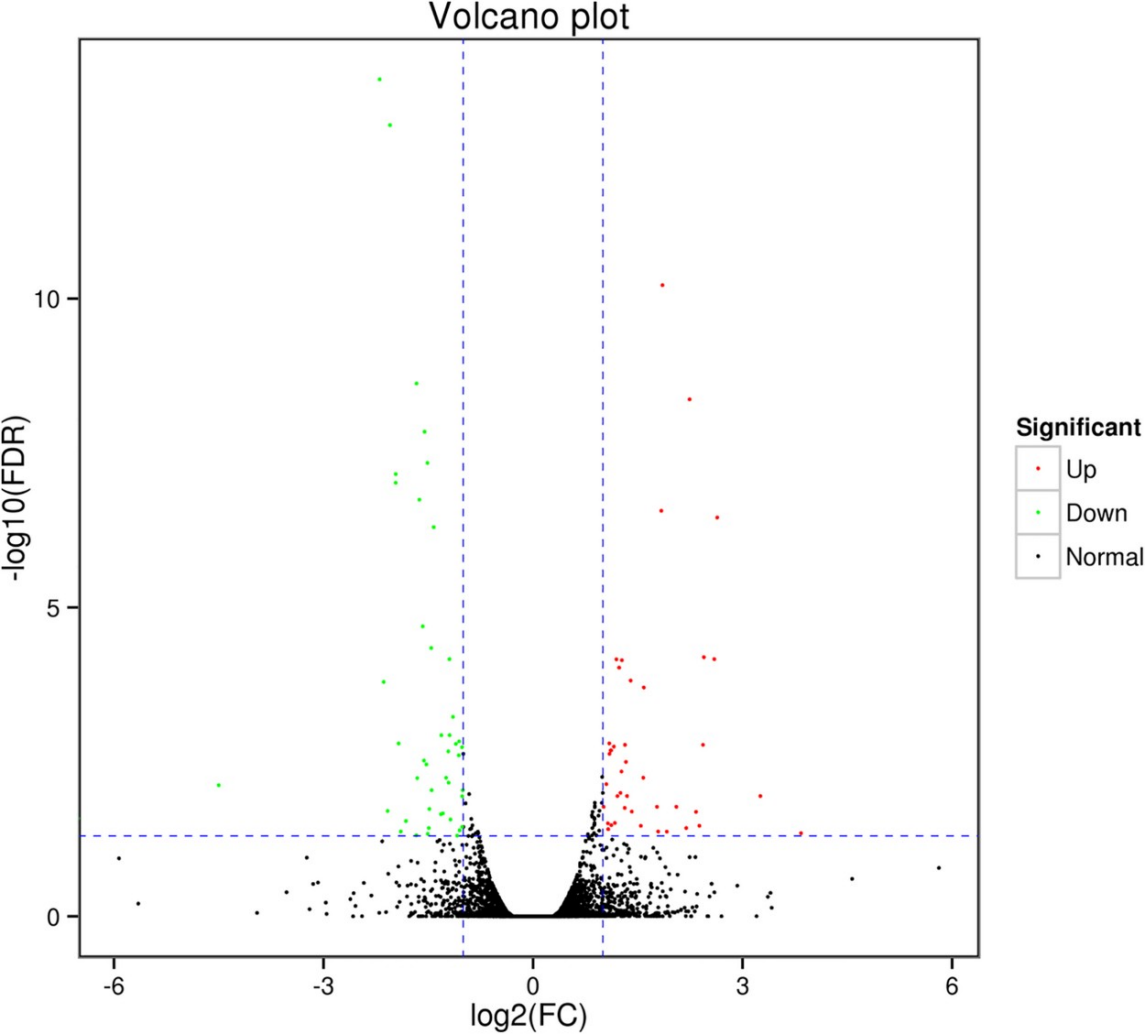
Volcano plot.

**Fig 2:**
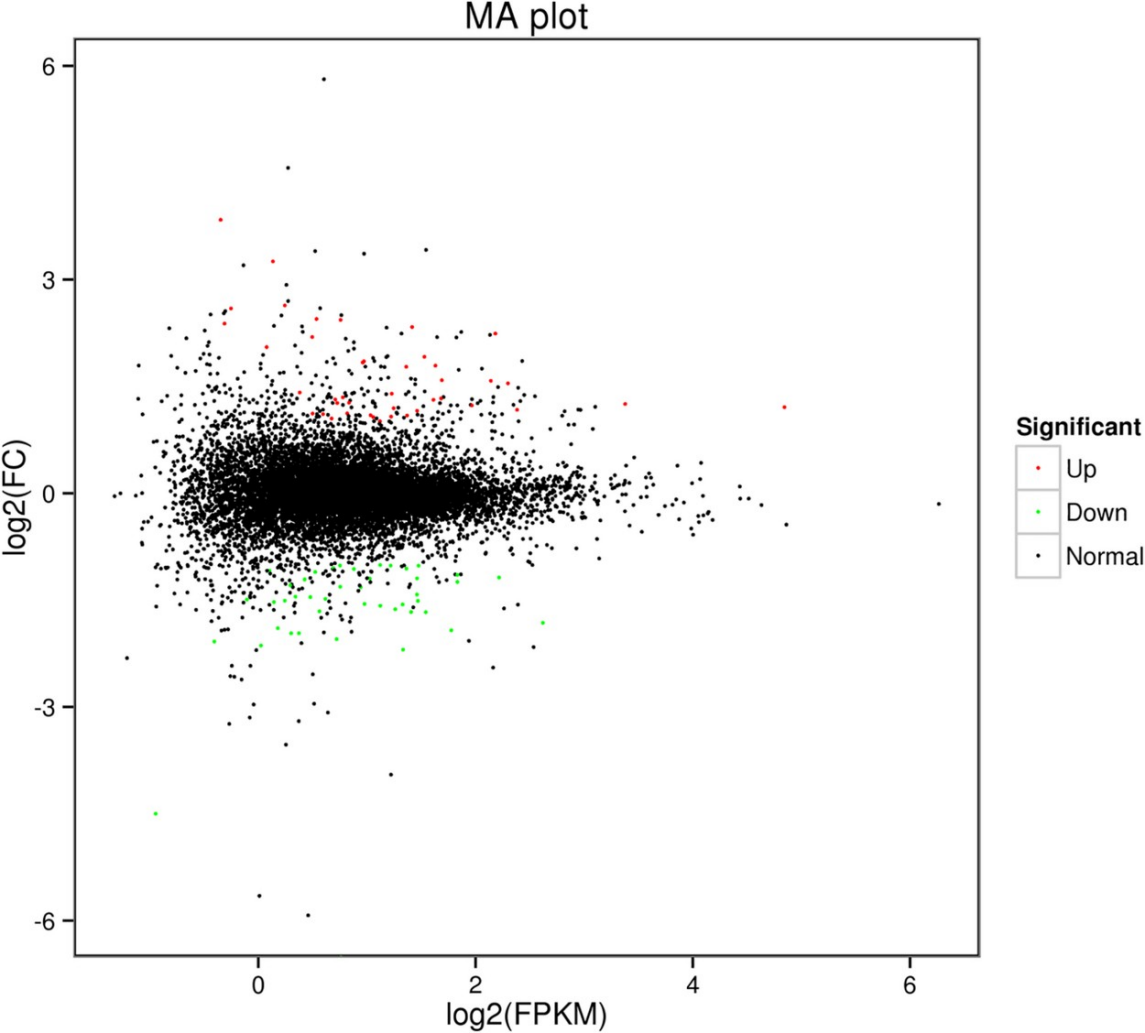
MA plot.

**Fig 3:**
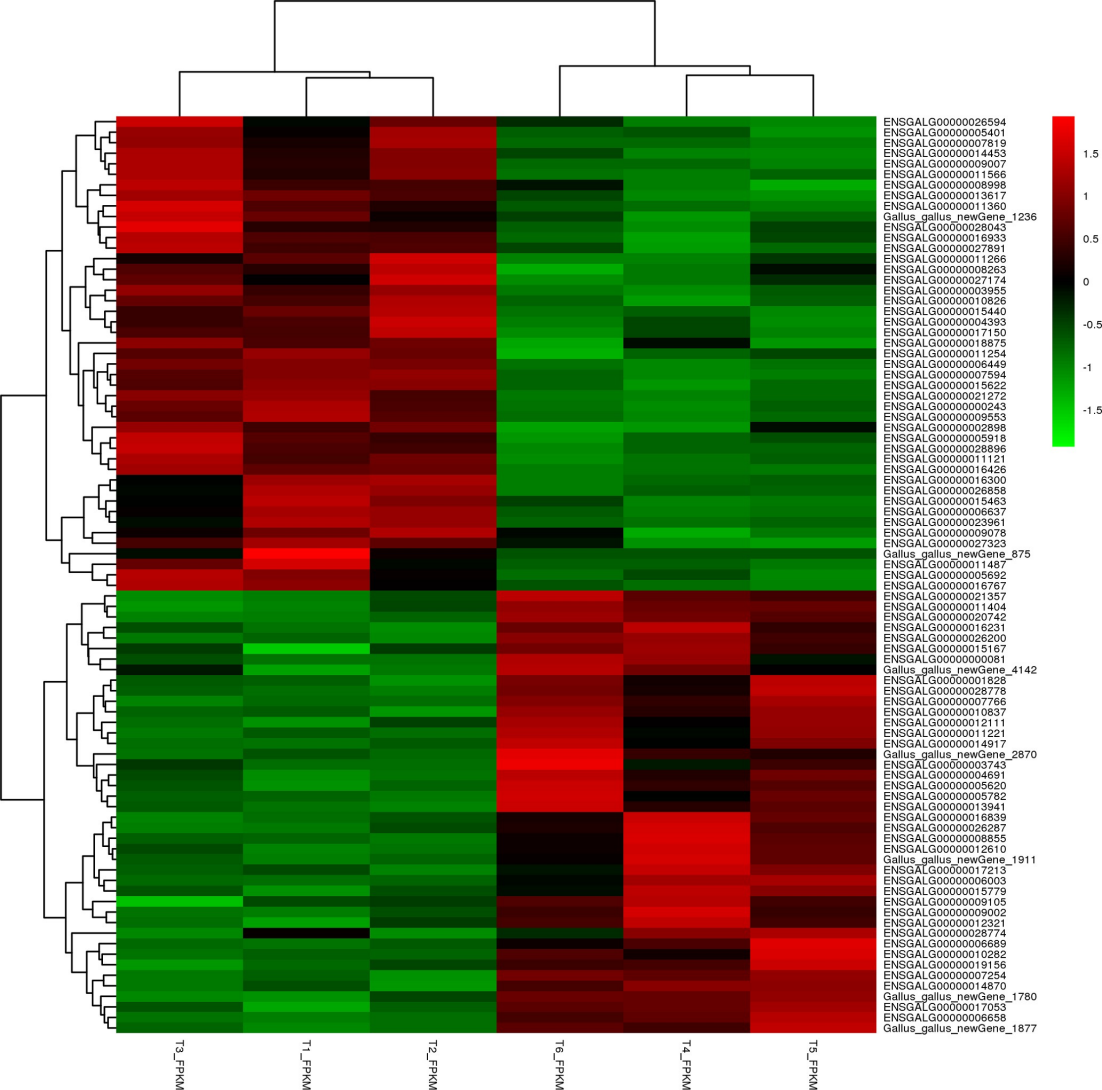
Cluster analysis of DEGs.

A total of 87 differentially expressed genes (DEGs) were identified by comparing the gene expression between the two groups (fold change, FC ≥ 2 and False Discovery Rate, FDR < 0.05). Compared to the slow-growing group (T1, T2, T3), the fast-growing group (T4, T5, T6) had 42 up-regulated genes and 45 down-regulated genes among these DEGs. Using a volcanic map (volcano plot), we can see the difference in gene expression level between the two groups and the statistical significance of the difference (Fig 1). Using the MA diagram (Fig 2), the expression level of the two groups and the overall distribution of the difference multiplier can be visually examined. A hierarchical cluster analysis was applied to the DEGs. We calculated the distance between the samples using the expression of different genes in each sample and determined the correlation between the samples. It was found that the same group of differentially expressed genes were clustered in the same cluster (Fig 3), which illustrated the accuracy and reliability of samples.

### GO enrichment and KEGG pathway analysis for DEGs

The DEGs were categorized into three main GO categories: biological process, cellular component, and molecular function. Among the 87 DEGs, 76 genes were annotated. In the biological process, the DEGs were significantly enriched in 6 items, and the number of enriched genes in the items was 7, 6, 4, 3, 7 and 3. The enriched genes and the name of each item are shown in Table 4.

**Table 4:** Significantly enriched biological process terms.

Term ID	Term	Count	P-Value	Genes
GO:0009792	embryo development ending in birth or egg hatching	7	0.001055	NLE1, WNT9A, KIAA1217, SLC35D1, SHROOM3, EYA1, MSTN
GO:0043009	chordate embryonic development	6	0.005054	NLE1, WNT9A,KIAA1217, SLC35D1, SHROOM3, EYA1
GO:0048706	embryonic skeletal system development	4	0.008883	WNT9A, KIAA1217, SLC35D1, EYA1
GO:1901654	response to ketone	3	0.027751	ABHD2, PPKAA2, MSTN
GO:0009790	embryo development	7	0.038256	NLE1, WNT9A, KIAA1217, SLC35D1, SHROOM3, EYA1, MSTN
GO:0044272	sulfur compound biosynthetic process	3	0.039303	GCLM, SLC35D1, HS3ST5

**Fig 4:**
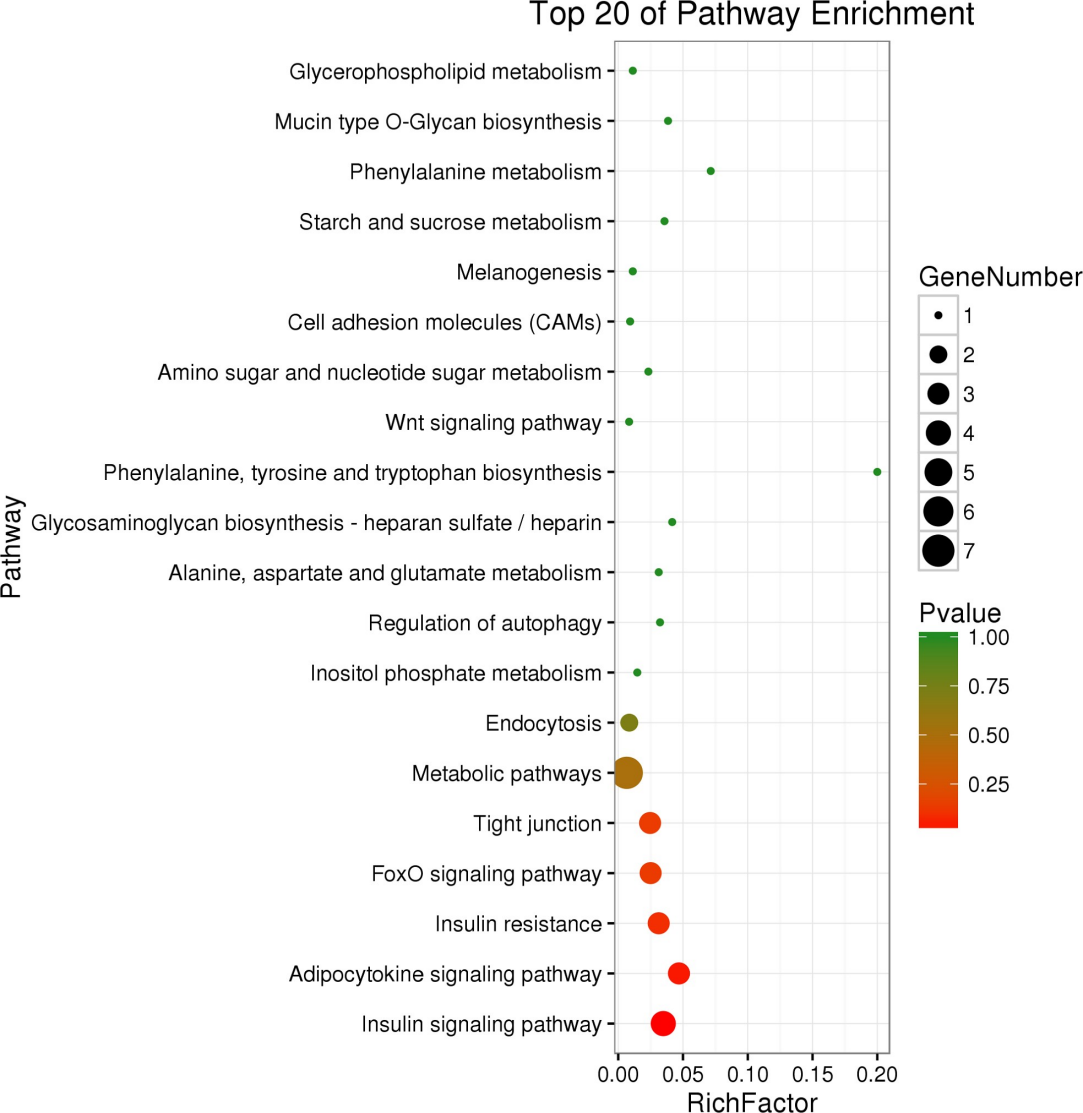
Top 20 genes of pathway enrichment.

The KEGG pathways of the differentially expressed genes are shown in Fig 4. The figure shows the first 20 pathways with the smallest P-values. Only two signaling pathways were significantly enriched (P-value < 0.05): the insulin signaling pathway and the adipocytokine signaling pathway (Table 5). Four genes were enriched in the insulin signaling pathway, and the adipocytokine signaling pathway was enriched in three genes.

**Table 5:** Significantly enriched pathway.

Term	Count	P-Value	Genes
gga04910:Insulin signaling pathway	4	0.021645	SH2B adaptor protein 2(SH2B2); protein kinase, AMP-activated, alpha 2 catalytic subunit(PRKAA2); protein kinase, AMP-activated, gamma 3 non-catalytic subunit(PRKAG3); insulin receptor substrate 2(IRS2).
gga04920: Adipocytokine signaling pathway	3	0.04416	protein kinase, AMP-activated, alpha 2 catalytic subunit(PRKAA2); protein kinase, AMP-activated, gamma 3 non-catalytic subunit(PRKAG3); insulin receptor substrate 2(IRS2)

### Validation of DEGs by qRT-PCR

**Fig 5:**
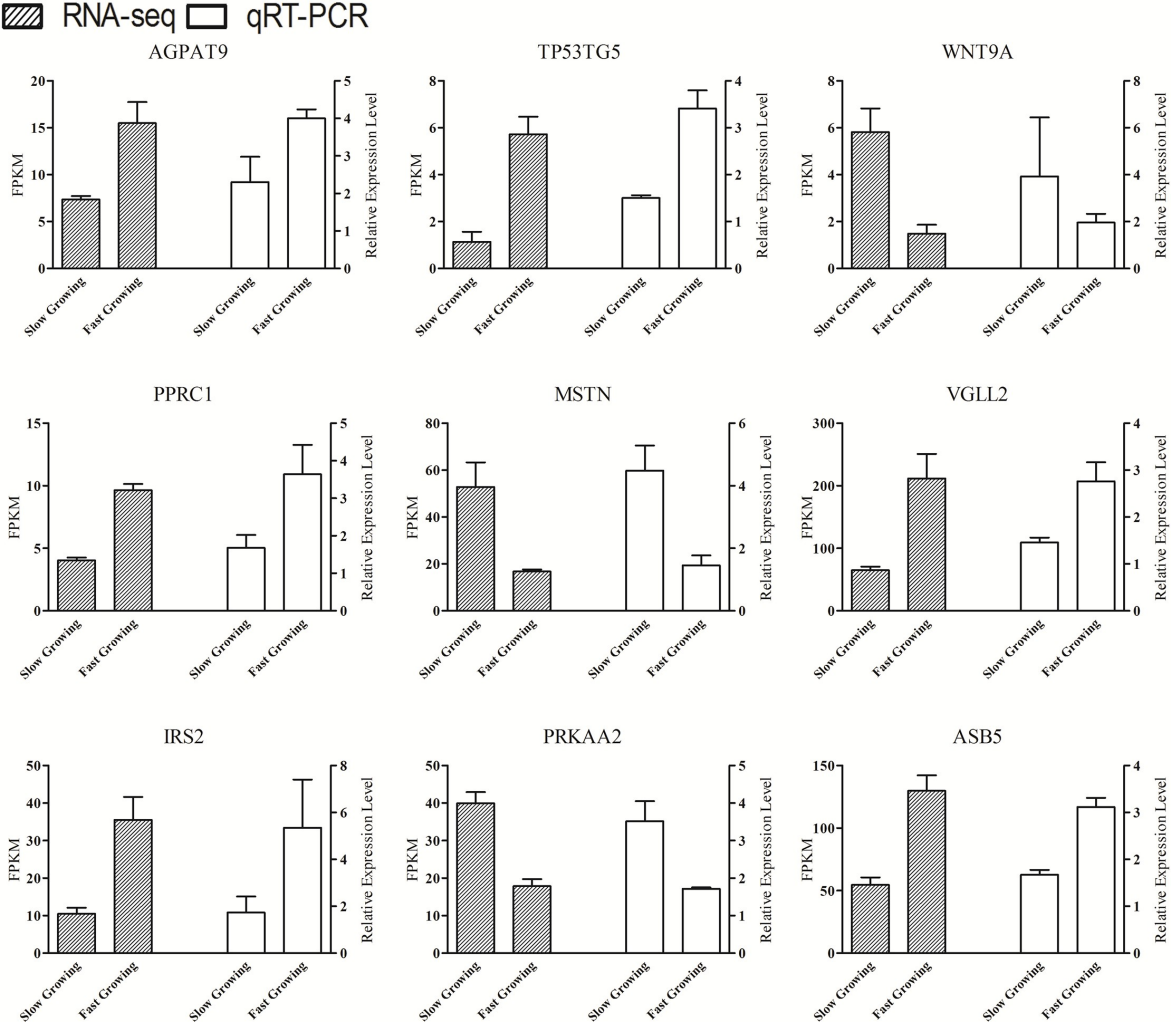
Expression level of nine DEGs detected by RNA-seq and qRT-PCR.

In our study, the expression of DEGs between the two groups was verified using quantitative real-time PCR (qRT-PCR). Nine DEGs obtained from RNA-seq were randomly selected for validation: AGPAT9, TP53TG5, WNT9A, PPRC1, MSTN, VGLL2, IRS2, PRKAA2 and ASB5. The results (Fig 5) showed that the expression trend of the DEGs between the fast- and slow-growing groups is consistent in qRT-PCR results, and this attests to the reliability of the sequencing data.

## Discussion

Chicken is widely purchased by consumers in the market because of its unique flavor and relatively reasonable price. The share of chicken in the market has also increased largely in recent years [28, 29]. Although some genes related to growth have been discovered, the specific mechanisms for growth and development are still unclear. In our study, RNA-seq in chickens with different growth rates was carried out. A total of 87 differentially expressed genes (DEGs) were identified by comparing the gene expression between the fast- and slow-growing groups. Many of the DEGs selected in the study are known for their impact on growth, development and meat quality, including MSTN, VGLL2, MYH1D, MYH1E, PRKAG3, and IRS2.

As a regulator of skeletal muscle growth, MSTN plays a key role in negatively regulating the growth and development of skeletal muscle and influencing the strength and quality of muscles [30–32]. In prior studies, researchers showed that Ross birds were significantly larger than the Illinois birds at all time-points from post-hatch day 7 to post-hatch day 35, and the difference was even more pronounced when the breast muscle mass was compared [33]. In the next study, MSTN expression was strongly detected in Illinois birds compared to the Ross birds at 21 days post-hatch in the breast muscle [34]. In this study, higher expression of the MSTN gene was detected in the slow-growing chicken group, and this was consistent with previous research studies of the gene function. In Drosophila, the nuclear protein Vestigial plays a crucial role in the development and patterning of the wing [35, 36]. Previous reports showed that one of the members of the Vestigial-like factors, VGLL2, can activate the TEAD family of transcription factors by physical interaction in *in vitro* assays [37]. Honda M et al. [38] further confirmed that VGLL2 formed a complex with TEAD1/4, which regulates the expression of muscle-specific genes in neonatal mouse muscle. A study showed that VGLL2 might act downstream of MyoD activation and is associated with skeletal muscle differentiation in chick myogenesis [39]. In this study, the transcriptome data showed that the expression level of the VGLL2 gene in the fast-growing chicken group was significantly higher than in the slow-growing group. This result indicated that the gene might have a positive effect on growth in chickens.

In recent years, with the progress of breeding, the growth rate of poultry has been significantly improved, but the meat quality has declined. The reason for the result may be that the selection of breeding will change the properties of muscle fiber, which is closely related to meat quality [40, 41]. Myosin heavy chain (MYH) isoforms are mainly expressed in the skeletal muscle of mammals and could define types of muscle fiber [42, 43]. Zuo et al. [44] found that the overexpression of miR-143 ultimately acts on the MYHC7 gene and controls the formation of slow fibers in swine. MYH1, an MYH family isoform, is found to be involved in the metabolism and development of skeletal muscle [45]. MYH1D and MYH1E, which are similar to the MYH1, were found to be significantly differentially expressed in the two groups. It could be inferred that the two genes influence the growth and development of the chicken by regulating the formation of different muscle fiber types. Previous studies showed that PRKAG3 was also related to meat quality [46, 47]. Shu et al. [48] found that PRKAG3 expression levels were significantly higher in glycolytic skeletal muscle than in oxidative skeletal muscle by transcriptome sequencing, but a higher content of oxidative (red) fibers in muscles can result in higher meat quality [49]. This suggested that high expression of the PRKAG3 gene may decrease meat quality. In this study, higher expression of the PRKAG3 gene was detected in the slow-growing chicken group; therefore, we deduced that the flavor of fast-growing chicken may be better than that of slow-growing chicken. According to traditional views, the growth-rate may be negatively correlated with meat quality. However the quality of meat is also related to species and environmental factors. Therefore, the actual quality of meat needs further study.

Many reports revealed that insulin resistance was associated with obesity both in animals and humans [50, 51]. The expression of the insulin receptor substrate 2 (IRS2) gene could alleviate insulin resistance [52]. Compared to the slow-growing group, higher expression of the IRS2 gene was detected in the fast-growing group in this study. To stay healthy and prevent insulin resistance, fast-growing chickens with more fat might increase the expression of the IRS2 gene using its own regulatory system.

The results of broiler breeding could increase growth rate and appetite and were also accompanied by excessive deposition of fat [53, 54]. Studies on FAM134B are mainly associated with disease, especially cancer [55–57]. There are also some studies on fat deposition from FAM134B in pigs [58, 59]. Yuan et al. [60] found that FAM134B mRNA levels in the subcutaneous fat were significantly higher in Jinhua pigs (a slow-growing breed) than those in Landrace pigs (a fast-growing breed) at 90 d. However, the result in this study was contrary to the previous study in pigs, which might be due to using different tissues and species. The AGPAT9 gene was also involved in the metabolism of fat [61, 62]. The expression of AGPA9 had the highest abundance in adipocytes compared to other tissues in female chickens [63]. In another study, chicks were fed (continuous ad libitum access to food), fasted (3 h food withdrawal), or refed (fasted for 3 h and refed for 1 h) before measuring the expression of the AGPAT9 gene. The results showed that the mRNA level of AGPAT9 was higher in the subcutaneous adipose tissue of fed chickens compared to fasted or refed chicks (P < 0.05). It was also greater in fed than refed chicks (P < 0.05) in abdominal adipose tissue [64]. In this experiment, the expression of AGPAT9 was significantly lower in the slow-growing group, which suggests that the fast-growing chickens might have a high fat content. As is known to all that high growth rate of animals generally results from a high appetite which could lead to fat deposition [65].

The growth of chickens is controlled by multiple genes through biological processes and different regulatory pathways. GO analysis showed that the DEGs were significantly enriched in embryo development ending in birth or egg hatching, chordate embryonic development, embryonic skeletal system development, tissue development, embryo development, tissue development and response to ketone in terms of biological processes. The KEGG pathway enrichment results showed that the first several pathways that were the most reliable were all related to growth and development. These were the insulin signaling pathway, adipocytokine signaling pathway, insulin resistance, FoxO signaling pathway, tight junction and metabolic pathways. However, only the first two pathways were significantly enriched (P < 0.05).

The insulin-signaling pathway was demonstrated to be involved in translation initiation, and the efficiency of the translation process directly affects the rate of protein synthesis [16]. Insulin, as an important member of the insulin signaling pathway, is the major regulator of the fasting-to-fed metabolic transition by altering substrate metabolism, promoting energy storage, and activating protein synthesis. In addition to its glucose and other metabolic properties, insulin can also stimulate cell [66, 67] and neuronal growth [68, 69]. The insulin signaling pathway was the most significantly enriched in the study, which suggested that substrate metabolism, activating protein synthesis and cell growth differed between the slow- and fast-growing groups. Another significantly enriched pathway was the adipocytokine signaling pathway. Adipocytokines are a type of cytokines synthesized and secreted by adipocytes, and they have a regulatory effect on inflammation, insulin sensitivity, and endothelial function [70, 71]. Three pathways, including the adipocytokine signaling pathway, were found to be significantly enriched (P < 0.05) for the targets of novel-mir-14 in the research of breast muscle in the Pekin duck [72].

Although the insulin resistance, FoxO signaling pathway, tight junction and metabolic pathways were not significantly enriched, we still thought that they played a decisive role in the different phenotypes of fast- and slow-growing chickens. Type 2 diabetes mellitus (T2DM) is characterized by impaired glucose intolerance and insulin resistance [72]. It is also well-known that lipid metabolism disorder is associated with development of insulin resistance and T2DM [73]. Obesity is characterized by insulin resistance and chronic low-grade inflammation [50]. FOXO3, which was found to participate in regulating the insulin and the IGF1 signaling pathway, played an important role in growth [74]. The FOXO family of proteins regulates atrophy, and insulin prevents cardiac muscle atrophy by inhibiting FOXO through a PKB/Akt-dependent pathway [51]. Six DEGs are involved in the FoxO signaling pathway in the RNA-seq of broiler chicken kidneys [13]. The insulin signaling pathway, adipocytokine signaling pathway, FoxO signaling pathway and tight junctions were also shown to be significantly enriched by Gao et al. [75] in chicken myocardial cells. In the KEGG analysis, Xue et al. [16] also identified pathways related to growth, namely, tight junction and insulin signaling pathways, of which the insulin signaling pathway was the most significantly enriched.

## Conclusions

This study systematically reveals the differentially expressed genes, significantly enriched items and KEGG pathways between fast- and slow-growing chickens, which could play an important role in the regulation of development in the chicken. The results further expand our understanding of the genes and their pathways associated with growth in the chicken. The experimental data provide a theoretical basis for improving the production performance of the Jinghai yellow chicken. It also provides reference data for revealing the molecular mechanisms of chicken growth.

## Supporting information

S1 Table Differentially expressed genes. (XLSX)

S2 Table Primers for qRT-PCR. (XLSX)

S3 Table The growth performance of chickens for RNA-seq. (XLSX)

S4 Table The overall growth performance of chicken population. (XLSX)

S5 Table The diet composition. (XLSX)

S6 Table The fold change of RNA-seq and qRT-PCR. (XLS)
